# Application of NeuroTrace staining in the fresh frozen brain samples to laser microdissection combined with quantitative RT-PCR analysis

**DOI:** 10.1186/s13104-015-1222-9

**Published:** 2015-06-20

**Authors:** Seico Benner, Masaki Kakeyama, Toshihiro Endo, Wataru Yoshioka, Chiharu Tohyama

**Affiliations:** Laboratory of Environmental Health Sciences, Center for Disease Biology and Integrative Medicine, Graduate School of Medicine, The University of Tokyo, 7-3-1 Hongo, Bunkyo-ku, Tokyo, 113-0033 Japan; Dept. Neurobiol. and Behav. Grad. Sch. Biomed. Sci, Nagasaki University, Nagasaki, Japan; Department of Pathophysiology, Tokyo University of Pharmacy and Life Sciences, Tokyo, Japan; Environmental Biology Laboratory, Faculty of Medicine, University of Tsukuba, Tsukuba, Japan

**Keywords:** LMD RT-qPCR, NeuroTrace staining, RNA transcript profiling

## Abstract

**Background:**

The heterogeneity of the brain requires appropriate molecular biological approaches to account for its morphological complexity. Laser-assisted microdissection followed by transcript profiling by quantitative determination has been reported to be an optimal methodology. Nevertheless, not all brain regions can be identified easily without staining, restricting the accuracy and efficiency in sampling. The aim of the present study was to validate whether fixation and staining treatments are suitable for quantitative transcript expression analysis in laser microdissection (LMD) samples. Quantitative RT-PCR was used to determine the absolute transcript expression levels and profiles of samples obtained from the hippocampal dentate gyrus from fresh frozen mice brain sections that had been fixed with ethanol and stained with NeuroTrace. The results were compared with those obtained from unfixed and unstained samples.

**Results:**

We found that the quantitative relationship of transcript expression levels between various housekeeping genes and immediate early genes was preserved, although the preparation compromised the yield of the transcripts. In addition, histological and molecular integrities of the fixed and stained specimens were preserved for at least a week at room temperature. Based on the lobe specific profiles of transcripts in the anterior and posterior lobes of the pituitary, we confirmed that no cross-contamination on transcription expressions occurred as a result of the fixation and staining.

**Conclusions:**

We have provided detailed information of the procedures on ethanol fixation followed by NeuroTrace staining on the absolute quantitative RT-PCR analysis using microdissected fresh frozen mouse brain tissues. The present study demonstrated that quantitative transcript expression analysis can be conducted reliably on stained tissues. This method is suitable for applications in basic and clinical studies on particular transcript expressions in various regions of the brain.

**Electronic supplementary material:**

The online version of this article (doi:10.1186/s13104-015-1222-9) contains supplementary material, which is available to authorized users.

## Background

Laser-assisted microdissection (LAM) has been established as a molecular biology tool optimal for obtaining specifically selected cells from non-homogeneous tissues, such as brain tissues. There are basically two LAM systems in the target isolation method: laser microdissection (LMD, or laser excision) and laser capture microscopy (LCM). The comparison of these two systems are not the direct aim of our present study, and a large body of literature has been published in biomedical research fields that provides data on nucleic acid and protein analysis in regions of interest (ROIs) using both approaches [1–4].

The quality of macromolecules retrieved from LAM specimens depends on the manner in which the tissues are treated before being subjected to LAM. It is a prerequisite that the targeted tissue is precisely distinguished from the adjacent areas and that the integrity of macromolecules in the dissected tissue is well preserved. Fresh frozen tissues cryosectioned for LAM provide a yield of macromolecules, such as transcripts, sufficient for subsequent analysis [1, 5]. Our recently reported protocol using RNA isolation and quantitative reverse transcription PCR (RT-qPCR) [6] enables researchers to stably quantify transcript expression levels in tissue sections in various brain regions with sizes >10,000 μm^2^ and thicknesses of 10–30 μm, estimated to contain several hundred neurons. This protocol is designed to be applied for a quantitative gene expression analysis of a small amount of samples by omitting the RNA refining steps and RNA amplification. In detail, the method excludes processes that may lose transcripts, such as washes. In addition, the amplification step is omitted to retain, as much as possible, the expression ratio of existing transcripts within a given tissue. These modified procedures attempt to minimize the loss of RNA samples while preserving the expression ratio of a variety of transcripts within a sample. Using this method, the amount of 18S rRNA and β-actin was shown to be proportional to the sample size, and to each other in samples of different sizes. The sensitivity and precision of this method have been demonstrated with the lower limit of sampling size corresponding to a single cell [6].

The challenge, however, is that not all tissue regions can be identified easily without fixation and staining, which restricts the efficient and reproducible sampling of ROIs for scientific investigation. Because fixation and staining of tissue sections enable high-resolution sampling of ROIs that are otherwise indistinguishable, there is a need for reliable techniques to conduct LAM-based molecular studies, which may also be useful for histological analysis. The prerequisite is that transcripts remain stable throughout these experimental manipulations and during the dissection procedure performed at room temperature, which has been the topic of interest in this research field [4, 5, 7, 8]. Another critical prerequisite for applying fixation and staining procedures in LAM-based molecular studies is that the expression ratio of the existing transcripts, the transcript expression profile, remains preserved after these treatments.

How different experimental manipulations during tissue preparation for varying LCM platforms affect the RNA quality has been one of the critical issues when combining microdissection with absolute quantitative RT-PCR for gene expression analysis. For example, Kerman et al. [8]. investigated the effects of staining on RNA integrity in fresh frozen brain tissue microdissected using LCM which melts adhesive plastic onto the tissue of interest by a low-power laser and lifts off the slide using an apposed cap. They have concluded that the RNA degradation level based on the 18S peak, analyzed by the 2100 BioAnalyzer, was a more reliable indicator of RNA quality than the 28S/18S ratio. They have also found that the RNA integrity number (RIN) significantly correlates with the ratio of 18S to the baseline, suggesting that the expression level of 18S would be a reasonable indicator for measuring RNA integrity. However, the effect of staining on transcript expression profile has not been studied. Grundemann et al. [7] demonstrated a protocol for a gene expression analysis from Nissl stained fresh frozen human and mouse brain tissues on polyethylene naphthalate (PEN) membrane slides using the UV-LMD (Leica LMD 6000), with varying concentrations of ethanol and aqueous staining solution, yet effects of fixation and staining on the RNA yield and its expression profile remained to be evaluated.

We considered the use of NeuroTrace, which is a widely used fluorescent Nissl stain for neural tissues applied for morphological and pathological investigations. Nissl staining has been proven to be inert for subsequent genetic applications [9], however, its detrimental effects have also been reported on cultured mouse plasma cells [10]. Although a quantitative transcription expression analysis has previously been conducted using the mouse hippocampal sections prefixed by transcardial perfusion with 4% paraformaldehyde followed by NeuroTrace staining [6], the extent of preservation of intact nucleic acids that was captured from the stained tissues has not been evaluated. Furthermore, the efficacy of neuronal staining methods on fresh frozen sections in combination with quantitative transcript expression level analysis remains unknown.

In this study, we aimed to assess the possible effects of fixation and staining on the expression levels of various transcripts. We performed quantitative gene expression analysis based on our previously developed LMD RT-qPCR protocol [6], using fresh frozen mice brains. Importantly, our primary aim was to accomplish an accurate quantification of the target transcript expressions in various brain ROIs. It was essential to validate whether staining could affect the expression profile of the tissue since this has not been evaluated previously. We used an LMD platform that employs a high-power laser to cut around the tissue of interest which then falls into a collecting cap by gravity. We selected polyphenylene sulfide (PPS) membrane frame slides which are excellent for extremely small samples, e.g. for single-cell dissection, with a very low autofluorescence compared to other standard membranes such as PEN membrane slides. The hippocampal dentate gyrus (DG) region of the mouse brain was selected to evaluate the impact of post-fixation with absolute ethanol followed by NeuroTrace staining, and the methodology was further validated using the pituitary gland. Besides housekeeping genes and neural marker Map2, expression levels of various immediate early genes (IEGs) were measured. IEGs are known to reflect cellular response, and are considered to play an essential role in brain functions such as neuronal plasticity, thus have been widely used in neuroscience research [11–13].

## Results

### Visualization of neurons by fixation and staining

Neuronal staining prior to LMD aids the visualization of boundaries of a specific brain region, which is a prerequisite for precise LMD procedures, and enables sampling at the single-cell scale. We examined the quality of NeuroTrace staining using our quick staining method.

Under the LMD microscope’s fluorescent light (LMG setting), NeuroTrace staining, in combination with ethanol fixation, clearly revealed identifiable neuronal somas, as observed in the region near the third ventricle and within the choroid plexus (Additional file 1: Figure S1 A_1_, A_2_). The regional boundaries of the CA1and DG in the hippocampus were distinct with NeuroTrace staining (the right side of Additional file 1: Figure S1 A_3_, B). Although the intrinsic fluorescence was observed under unstained conditions (e.g., the left side of Additional file 1: Figure S1 A_3_), under higher magnification, only stained tissue displayed a visualization quality at a resolution optimal for microdissection at a single-cell scale (Additional file 1: Figure S1 B_2_, B_3_).

### Effects of ethanol fixation and NeuroTrace staining on transcript expression levels

**Figure 1:**
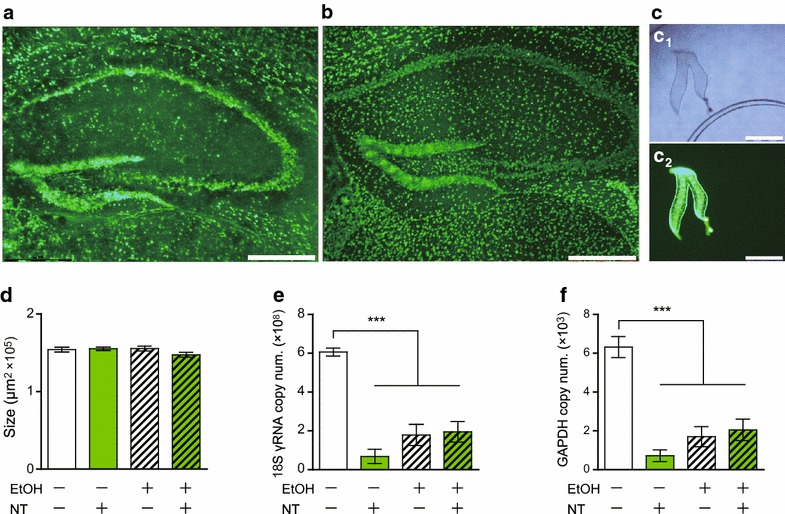
Visualization of neurons under a fluorescence radiated field, of a mouse hippocampus stained with NeuroTrace **a** without fixation and **b** with ethanol fixation. **c** A single dentate gyrus (DG) was collected, **d** 150,000 μm^2^ × 20 μm in volume, for the following each experimental condition: unfixed and unstained, NeuroTrace stained without ethanol fixation, ethanol fixation without staining, and ethanol (EtOH) and NeuroTrace (NT) treated. Transcript levels of **e** 18S rRNA and **f** GAPDH mRNA. Values are expressed as copy number of transcripts. *Bars* indicate mean ± SEM (*n* = 6 each). One-way ANOVA followed by Tukey post hoc test. *p < 0.05, **p < 0.01, ***p < 0.001. *Scale bars* 310 μm.

Ethanol fixation prior to NeuroTrace staining of the hippocampal region resulted in clearer staining than that obtained without fixation (Figure 1a, b). Using LMD, the hippocampal DG (Figure 1c) was microdissected as ROIs for transcript expression level analysis. The microdissected size of each sample was adjusted to approximately 150,000 μm^2^ (Figure 1d), which is estimated to have thousands of neurons. RNA extraction and reverse transcription were performed on the day after fixation and staining. For fragments 150,000 μm^2^ × 20 μm in volume.

For comparison between specimens that were fixed and stained under different conditions, consecutive cryosections were alternately placed on separate steel-framed PPS membrane slides under the following treatment procedures: ethanol fixation only, NeuroTrace staining only, and a combination of both. The transcript expression levels of the housekeeping genes 18S rRNA (Figure 1e) and GAPDH (Figure 1f) were significantly affected by ethanol fixation and/or NeuroTrace staining treatments: the percentages of transcripts retained for each experimental setting were as follows (mean ± SEM): NeuroTrace staining without ethanol fixation, 11 ± 7% (18S rRNA) and 11 ± 5% (GAPDH); ethanol fixation without NeuroTrace staining, 29 ± 9% (18S rRNA) and 27 ± 8% (GAPDH); a combination of ethanol fixation and NeuroTrace staining, 32 ± 9% (18S rRNA), 32 ± 9% (GAPDH). Based on these results, we performed the rest of our experiments by simply comparing the unfixed/unstained samples with the fixed/stained samples since staining without fixation deteriorated the samples, making them inappropriate for histological observations.

### Effects of time elapsed after fixation and staining on transcript expression levels

We aimed to determine whether the time elapsed after ethanol fixation and NeuroTrace staining treatment affects the quality of retrievable transcripts, because RNA degradation was suspected to occur over time. On the 8th day post staining, the hippocampal DG region (approximately 150,000 μm^2^) was microdissected and reverse transcribed (hereafter referred to as Day 8 samples) for comparative analysis with those that were reverse transcribed on the day after the staining (Day 1 samples). Day 1 and Day 8 samples were collected from the same cryosections to evaluate the effect of time under identical fixation and staining conditions. The side (left/right) of DG samples was counterbalanced between the Day 1 and Day 8 samples.

**Figure 2:**
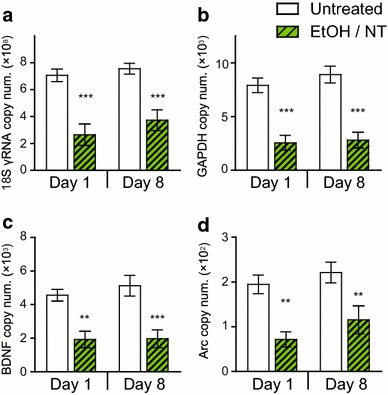
Transcript levels of **a** 18S rRNA, **b** GAPDH, **c** BDNF, and **d** Arc in samples that were reverse transcribed on Day 1 (*n* = 6 each) and Day 8 (*n* = 7 each) after the ethanol and NeuroTrace (EtOH/NT) treatment, or unfixed and unstained (Untreated). The collected DG samples were 150,000 μm^2^ × 20 μm in volume per sample. Values are expressed as copy number of transcripts. *Bars* indicate mean ± SEM. Two-way ANOVA followed by Bonferroni post hoc test. **p < 0.01, ***p < 0.001 for the treatment effect.

There was no significant difference between Day 1 and Day 8 samples for the transcript levels of the housekeeping genes (Figure 2a, b) and IEGs (Figure 2c, d) in the untreated samples as well as samples that were fixed and stained. Consistent with the previous result (Figure 1e, f), the fixation and staining effect was statistically significant: 18S rRNA, [*F*(1, 22) = 42.32, *p* < 0.0001] (Figure 2a); GAPDH, [*F*(1, 22) = 61.01, *p* < 0.0001] (Figure 2b); BDNF, [*F*(1, 22) = 28.99, *p* < 0.0001] (Figure 2c); and Arc, [*F*(1, 22) = 21.77, *p* = 0.0001] (Figure 2d). In contrast, the time effect was not significant: 18S rRNA, [*F*(1, 22) = 1.54, *p* = 0.23] (Figure 2a); GAPDH, [*F*(1, 22) = 0.74, *p* = 0.40] (Figure 2b); BDNF, [*F*(1, 22) = 0.32, *p* = 0.58] (Figure 2c); and Arc, [*F*(1, 22) = 2.07, *p* = 0.16] (Figure 2d). In general, approximately one-third of the transcripts were estimated to be retained after fixation and staining in both Day 1 and Day 8 samples (Table 1).

**Table 1:** The values of the transcripts retained in samples reverse transcribed on Day 1 and Day 8 post treatment

	Day 1		Day 8	
	Untreated	EtOH/NT	Untreated	EtOH/NT
*18S rRNA*	100 ± 7	37 ± 11***	100 ± 5	49 ± 10***
*GAPDH*	100 ± 9	32 ± 9***	100 ± 9	32 ± 8***
*β*-*actin*	100 ± 13	26 ± 8***	100 ± 12	29 ± 9***
*Map2*	100 ± 9	43 ± 10**	100 ± 11	45 ± 11***
*cFos*	100 ± 15	57 ± 13*	100 ± 11	56 ± 13*
*Arc*	100 ± 11	37 ± 9**	100 ± 11	52 ± 14*
*BDNF*	100 ± 6	34 ± 8***	100 ± 12	38 ± 10**
*TrkB*	100 ± 12	35 ± 11**	100 ± 6	50 ± 11**

* *p* < 0.05, ** *p* < 0.01, *** *p* < 0.001 by Student’s *t*-test between untreated samples and ethanol-fixed, NeuroTrace-stained samples (EtOH/NT) samples. There were no significant differences between the transcript retained values (%) of the samples reverse transcribed on Day 1 and Day 8 after the treatment, for any of the quantified genes

The histological condition of the staining in the tissues was confirmed to be preserved for over a week at room temperature (12-day images are shown in Additional file 2: Figure S2 A–C), and its quality was improved with ethanol fixation (Additional file 2: Figure S2 D). The stained tissue sections on the slides were kept in a shaded slide box and protected from light, with the exception of occasional short-period observations under an LMD microscope. The stain gradually wore out after a month of storage under the above-mentioned conditions.

### Correlation of transcript expression profiles

**Figure 3:**
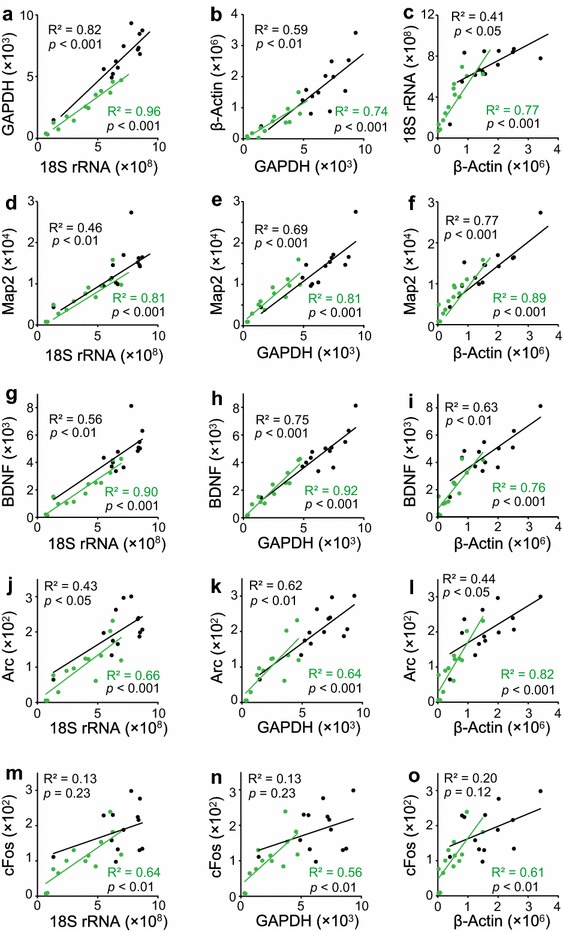
**a**–**c** Correlation of the expression levels between the housekeeping genes in the hippocampal DG region of unfixed and unstained (*black*) and ethanol-fixed and NeuroTrace-stained (*green*) tissue sections: **a** GAPDH vs. 18S rRNA, **b** β-actin vs. GAPDH, and **c** 18S rRNA vs. β-actin. Correlation between the expression levels of Map2 and **d** 18S rRNA, **e** GAPDH, and **f** β-actin. Correlation between the expression levels of BDNF and **g** 18S rRNA, **h** GAPDH, and **i** β-actin. Correlation between the expression levels of Arc and **j** 18S rRNA, **k** GAPDH, and **l** β-actin. Correlation between the expression levels of cFos and **m** 18S rRNA, **n** GAPDH, and **o** β-actin. Values are expressed as copy number of transcripts. Both untreated (*n* = 13) and treated (*n* = 13) samples were reverse transcribed on Day 1 and Day 8 after the treatment.

We examined whether the quantitative relationship of transcript levels among various housekeeping genes and IEGs was preserved, even though the amount of transcripts retained decreased as a result of fixation and staining. A significant correlation was found between the transcripts of various housekeeping genes (Figure 3a–c) as well as between Map2 (Figure 3d–f), BDNF (Figure 3g–i), Arc (Figure 3j–l), and cFos (Figure 3m–o). These correlations were found in both untreated and treated samples, indicating that the transcript profile was well preserved despite the significant loss of total transcripts after the fixation and staining. Importantly, significant correlations between various transcripts also remained in the samples used in the cryosection thickness analysis, for both untreated and treated samples (Additional file 3: Figure S3).

### Effects of cryosection thickness on transcript expression levels

Cryosectioning at a thickness of 20 μm was suspected to expose the cytoplasmic content of a large number of DG cells to fixation and staining fluids, because their soma diameter is approximately 10 μm. Thus, it was hypothesized that increasing cryosection thickness would result in greater proportions of intact cells than the thinner sections, therefore improving the retrieval rate of transcripts. However, it was found that no significant improvement was attained for the percentage of transcripts retained by increasing the cryosection thickness ranging from 20 to 40 μm (Additional file 4: Figure S4 A–D), as assessed by two-way analysis of variance (ANOVA): 18S rRNA, [*F*(2, 38) = 0.61, *p* = 0.55]; GAPDH, [*F*(2, 37) = 2.25, *p* = 0.12]; β-actin, [*F*(2, 38) = 2.26, *p* = 0.12]; and Map2, [*F*(2, 38) = 3.06, *p* = 0.06]. In contrast, the effect of treatment, i.e., fixation and staining, was statistically significant, consistent with the results from Figure 1: 18S rRNA, [*F*(1, 38) = 33.01, *p* < 0.0001]; GAPDH, [*F*(1, 37) = 42.46, *p* < 0.0001]; β-actin, [*F*(1, 38) = 39.62, *p* < 0.0001]; and Map2, [*F*(1, 38) = 28.20, *p* < 0.0001]. There was no interaction of thickness and treatment.

### Effects of duration of fixation on transcript expression levels

To evaluate whether a shorter time of ethanol fixation improves the amount of transcripts retained, unfixed or unstained samples were compared with those fixed in ethanol for 10, 30, and 60 s. There seemed to be a decreasing trend of transcription levels from the samples fixed for 10 s to samples fixed for 30 s or longer, yet the effect was insignificant (Figure Additional file 5: S5 A–C). The fixation time had no apparent drawback regarding the quality of the staining (Additional file 6: Figure S6).

### Application of the method to pituitary gland analysis

We examined whether cross-contamination of transcripts between the adjacent areas was observed in the present fixation and staining protocol because there was a concern for a flow-out of molecules caused during the fixation and staining process. If the transcripts had flowed out from their original positions and adhered to adjacent sections, it would defeat the entire purpose of the microdissection.

**Figure 4:**
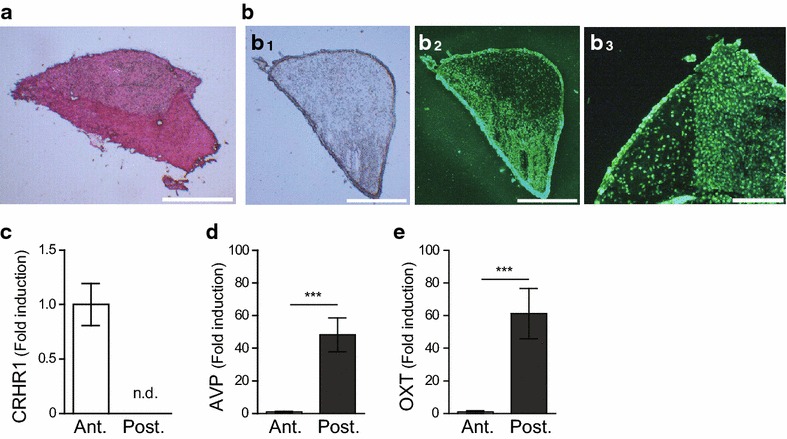
**a** H & E-stained pituitary cryosection on a silane-coated microslide. **b** Ethanol-fixed and NeuroTrace-stained pituitary sections under (**b**_**1**_) bright field and (**b**_**2**_, **b**_**3**_) fluorescence radiated field. **c**–**e** Comparative analysis between the anterior and posterior pituitary showing specific transcript expression profile for each region. **c** corticotropin-releasing hormone receptor 1; CRHR1, **d** vasopressin; AVP, and **e** oxytocin; OXT. The transcript levels in the posterior pituitary values (*n* = 8) were expressed as fold change compared to the anterior pituitary values (*n* = 9) and after normalization to GAPDH. *Bars* indicate mean ± SEM. *Asterisks* (***p < 0.001) indicate statistically significant difference between samples collected from the anterior and posterior pituitary glands, as assessed by Student’s *t*-test. *Scale bars* **a**, **b** 310 μm, and **c** 100 μm.

To examine this possibility, the mouse pituitary gland was cryosectioned, ethanol fixed, stained using NeuroTrace to visually identify the anterior and posterior tissues, and separately collected by LMD for the determination of the expression of subregion-specific transcripts. Pituitary glands are commonly stained with H & E to distinguish the anterior pituitary from the posterior pituitary via darker and paler staining, respectively (Figure 4a). After NeuroTrace staining, the boundary between the anterior and posterior pituitary became distinctive under fluorescent light, and the staining characteristics were similar to those of the H & E staining (Figure 4b_1_–b_3_). NeuroTrace-labeled anterior and posterior pituitary tissues were collected by LMD. The expression levels of region-specific transcripts were quantified to evaluate whether transcript profile specificities were preserved after the treatment involving ethanol fixation and NeuroTrace staining.

Transcript expression of corticotropin-releasing hormone receptor 1, which is specifically expressed in the anterior pituitary, was absent in LMD samples dissected as the posterior pituitary (Figure 4c). Both vasopressin and oxytocin transcripts are transported from the hypothalamus to the posterior pituitary via an axonal transport mechanism [14–16]. We found that there was approximately a 50-fold difference in the transcript levels of vasopressin (Figure 4d) and oxytocin (Figure 4e), which were significantly higher in the posterior pituitary than the anterior pituitary.

## Discussion

Microdissection of tissue sections under a laser-assisted microscope has been used to study physiological functions focusing on the morphology and its related gene expression in ROIs. We believe that the present study is the first to provide information on the preparation of fresh frozen brain specimens using NeuroTrace for LMD combined with absolute quantitative RT-PCR. This method paper aimed to solidify and extend our previous study [6], to describe the RNA extraction protocol in much further detail, and to confirm the validity of the staining applied to the LMD-RTqPCR method by showing the effect of staining on the expression ratio of various genes. In addition, in the previous study, paraformaldehyde-perfused samples were used for NeuroTrace staining, whereas this study focused on the use of the fresh frozen samples for the benefits mentioned in the section below. Here, we have demonstrated that our staining protocol allows differentiation of brain regions without disrupting the transcript expression profile of the treated specimens.

As one of the limitation in this study, we were unable to determine the quality and the exact amount of RNA obtained from the microdissected hippocampal region. This is due to the minute amount of total RNA, in addition to the solution constituents in which RNA was extracted in, both unsuitable for analysis by BioAnalyzer or gel electrophoresis. However, an absolute quantitation method using standard curves and two-step RT-qPCR enabled us to determine the percentage of transcripts retained from a specified sample size. This permitted the evaluation of the transcript expression ratio of various genes. The reason why fixation decreases the amount of transcripts retained requires further investigation, however, we have demonstrated that the transcript expression profiles of tissue sections approximately 20 μm in thickness were generally well preserved even after ethanol and NeuroTrace treatment, indicating that these tissue samples can be used for the quantitative analysis of transcript levels in a region- and cell-specific manner. It was noted, however, no significant correlations were found for the c-Fos transcript expression relative to the house keeping genes. This is presumably due to the low abundance of cFos transcripts compared to the housekeeping genes. In addition, c-Fos expressing cells are found to be sparsely distributed in hippocampal DG region based on immunohistochemistry images in the literature, which could have resulted in variability of c-Fos transcript expressions among different brain slices.

### Use of fresh frozen tissues and ethanol for staining

Fresh frozen or snap-frozen tissues have been considered optimal for the biochemical analysis of macromolecules [4, 17, 18]. For protein analysis, cryosectioning of fresh frozen brain tissues, without chemical fixation, was demonstrated to be resistant to degradation for up to 6 months when stored with a desiccant under ambient laboratory conditions [19]. For DNA analysis, the evaluation of ethanol fixation and H & E staining for frozen fresh biopsy tissue sections demonstrated that samples may be stored at room temperature for 4 years without DNA degradation [20]. Furthermore, fresh frozen tissues are considered as a reliable source of high-quality RNA [5, 21, 22]. It has been shown that RNA can be stably preserved in unfixed fresh frozen specimens, and it has been suggested for use in biobanking [23] for diagnostic testing and research. In contrast, RNA recovery from formalin-fixed and paraffin-embedded tissues is compromised because of the action of RNases, the effect of formalin on nucleic acids, and the influence of other chemicals on tissues during preservation and fixation [24, 25].

In terms of fixation, LMD samples from fresh frozen testis tissues fixed in ethanol yield high-quality RNAs consistently; fixation in acetone or ethanol provided the best morphology [26]. Similarly, for brain samples, fixation by ethanol is superior to formalin for preserving RNA integrity suitable for expression profiling of brain tissues by LCM [27]. Qin et al. [28] have previously demonstrated that a fresh frozen and ethanol fixed hippocampal DG granule cell sample yielded a transcript expression profile comparable to that of a non-fixed cell sample, and was more reliable compared to the RNA retrieved from paraformaldehyde-fixed, paraffin embedded tissue sample. Therefore, ethanol is considered to be a suitable fixation solution for fresh frozen brain tissues although staining effects on brain samples have not been previously reported. We evaluated the validity of applying our recently developed RT-qPCR method [6], which has been shown to be effective and reliable for the absolute quantification of transcript expressions in unstained samples, on fixed and stained brain tissue samples. The described method also is an advantage over previously described methods due to its technical simplicity; it requires no washes during RNA extraction and no RNA amplification step.

### Labeling cells for LMD applications

Examples of immunohistochemistry-guided LMD/LCM couple to microarray analysis is abundant in the literature, successfully demonstrating differential gene expression between differently labeled cells [29–33]. However, microarray requires a relatively high starting concentration of the RNA samples, amount impossible to obtain from the small number of cells from LMD-derived samples, thus requiring an amplification step. Furthermore, information regarding the effect of immunohistochemistry on gene expression profile is still limited [10], especially for brain tissues. For the current study’s purpose, optimization of the fixation and staining procedures is mandatory to preserve transcripts in tissue sections for RT-qPCR based transcript expression profiling specific to the anatomically defined ROIs in the brain. The preparations for labeling LAM specimens combined with the subsequent molecular biology analysis have already been addressed in a different context. For immunolabeling, Brown and Smith [34] developed an optimal method coupled with microdissection for transcriptome analysis, and demonstrated that immunolabeling in high salt buffer preserves RNA integrity of ethanol-fixed brain tissue. Combining this technique with a PALM MicroBeam system (Zeiss), they succeeded in isolating dopamine neurons [35]. Additionally, the benefits of rapid immunohistochemistry staining protocols in combination with LMD analysis have been described in previous studies [36–38]. For example, Fends et al. [38] demonstrated a rapid immunostaining technique for fresh frozen sections using tissues from neoplasmic lymph nodes, the breast, and the prostate, in combination with LCM, which allowed the recovery of high-quality mRNA. As to paraffin embedded human cortical neurons, Pitcher et al. [37] reported an effective immunohistochemistry protocol for quantitative analysis of protein and RNA expressions using LCM (PALMRobo).

While immunolabeling is a highly effective method for identifying specified molecules, non-specific neuron staining methods can instead be applied for the purpose of identifying different brain regions. However, the optimal protocols and the possible effects of fixation and staining for the subsequent LMD analysis have not been evaluated using fresh frozen brain tissues. Using brain tissues preperfused with RNase-free phosphate-buffered saline, Vincent et al. [39] described optimized conditions for quantifying histologically stained mouse hippocampal neurons using LCM followed by RT-qPCR, and showed that the total RNA yield correlated with the increase in laser-captured area, and that similar quantities of total RNA were retained in the three staining methods (i.e., NeuN immunohistochemistry, nuclear fast red, and hematoxylin). With several technical and procedural differences, the present study is similar to those reported previously, however, our present study demonstrated that ethanol fixation and NeuroTrace staining accurately maintain the proportionality of expression of various transcripts. Conventional Nissl-based staining methodologies such as the Cresyl violet staining, require numerous procedural steps, in particular a series of dehydration steps, with each step requiring considerable time. A direct immunofluorescence method in general requires fewer steps and less time, and here we have demonstrated that NeuroTrace staining, with minor modifications from our previous study [6], can be conducted in a single 1-min step. We also showed that no cross-contamination occurred as a result of the fixation and staining. These findings are thus particularly relevant for investigating brain subregions that are difficult to identify without labeling.

### Effects of fixation and staining on RNA stability

All of our evaluations were made by comparing LMD fragments having the same size obtained from the ROI of the identical brain area (hippocampal DG region) of mice treated under the identical experimental conditions. In particular, all the procedures, including microdissection, RNA extraction, reverse-transcription, and qPCR analysis were performed on the same day, which allowed precise evaluation of the impact of fixation and staining on the expression levels and ratios of various genes.

The timing of fixation and staining prior to the collection of tissue fragments by LMD must be rigidly regulated if there is a time-dependent RNA degradation during fixation and staining. Previously, Clement-Ziza et al. [40], have concluded that at least 90-min is granted to perform LCM experiments without RNA degradation post fixation and staining with ethanolic solutions of cresyl violet and eosin Y. This study showed that RNA integrity in both untreated samples or fixed and stained samples was preserved at room temperature for over a week, therefore a reasonably wide window of time is provided for LMD sampling post staining. RNA degradation could occur in fixed or stained samples during storage at room temperature if complete dehydration was not achieved. Since detectable progressive degradation did not occur in a week’s time, storage condition was not considered to be a critical factor in the quantitative transcript expression analysis.

No progressive loss in the amount of transcripts occured within the 1- to 8-day time frame post fixation and staining (Figure 2). This provides an experimenter sufficient time to microdissect a necessary number of samples. We consider this finding has practical value, since microdissecting all ROIs for multiple experimental groups, with sufficient n numbers to reach a statistically satisfactory conclusion may take several days, especially for those who must share the LMD apparatus with multiple users. In order to minimize the sample-to-sample variability that may occur in the process of RNA extraction and RT-qPCR, it is essential to conduct the RNA extraction and RT process all at the same time (i.e., using the same master mix for all samples), instead of conducting them sample-by-sample subsequently after each microdissection.

In addition, we have found that shortening the duration of fixation had a minimal effect on the percentage of transcripts retained, at least if fixation was performed within 60 s. The rate of diffusion of fixatives is assumed to be 1 h per mm of tissue thickness [41], calculated to take approximately 60 s for a 20-μm-thick section for the penetration into the tissue and the occurrence of chemical reactions.

As addressed in the methods section, tissue dryness is a critical issue for RNA integrity. Using an air dryer, we have demonstrated that the percentage of transcripts retained remained constant for a week. The low percentage of transcripts retained was not likely caused by moisture during or after the application of staining solution to fixed samples since the rate of transcript loss did not differ between the stained and unstained samples (Figure 1).

### Effects of cryosection thickness

In our study, the level of transcripts retained did not improve with increased cryosection thicknesses. This is presumably due to the inefficiency of LMD of a thicker specimen compared with a thinner specimen (Additional file 3: Figure S3). As a technical finding, we have found that a section thickness of 20 μm is an optimal thickness to work with. Attempting to cut sections with thicknesses over 20 μm frequently required more than a single trial, presumably due to an insufficiency in the laser power and aperture. When LMD was unsuccessful in achieving the complete dissection of ROIs in a single attempt, multiple laser beam irradiations were applied, which may have caused shaving of the rims. This presumably contributed to a lower number of collected cells than was originally intended. The optimal cryosection thickness should be decided upon aspects such as tissue intactness in terms of the laser dissection process, and the retrieval rate of transcripts post fixation and staining. Under our experimental conditions, 20 μm is recommended.

## Conclusion

A large body of literature has been published utilizing LAM technology, and there are high demands for technical investigations on improved methods for isolating ROI fragments and for the determination of transcripts in each fragment. In this study, we have studied the effects of fixation and staining procedures on the quantitative and qualitative characteristics of the transcript in the tissue fragment obtained from fresh frozen brain tissue samples, using LMD microscopy. The previous reports on in situ or the immunohistochemistry application to the LMD based gene quantification techniques are definitely useful, if the target cells or molecules have already been determined. However, in the case where the target molecules have not been revealed, and when one’s aim is to evaluate the expression profile of various transcripts in a targeted brain region rather than in a specific cell type, a simple neuron staining protocol is desirable. The proposed fixation and staining procedures are quick and simple. Also, histological and molecular integrities of the fixed and stained specimens can be preserved for at least a week at room temperature. Using the conditions we have verified on fixation and staining, this method can be applied to basic and clinical studies.

## Methods

### Brain sample preparation

Pregnant C57BL/6 mice were purchased from CLEA Japan (Tokyo, Japan), and their naïve female offspring (10 weeks old) were sacrificed and whole brains were collected and immediately frozen by burying them in powdered dry ice for 10 min. Brains were initially stored at −80°C, and transferred to −20°C on the day before sectioning on a cryostat (CM3050, Leica Microsystems K.K., Tokyo, Japan). The brains were subjected to cryosectioning at a thickness of 20–40 μm and then placed on steel-framed PPS membrane slides (Leica Cat. No.11505268). Continuous cryosectioned slices were alternately placed on the slides for different experimental conditions (untreated or fixed and/or stained), to minimize sample variations. The hippocampal DG, 150,000 μm^2^ × 20–40 μm in volume per section, was collected using an LMD microscope (model, LMD7000, Leica Microsystems). When RNA yields were compared between non-fixed and fixed tissues, the non-fixed tissues were microdissected at the same brain region (hippocampal DG), the size of which is equal to that of fixed or stained tissues (150,000 μm^2^). Samples from sequential recut of the same tissue were used to compare each condition: fixation, staining, elapsed time from the fixation and staining until RNA extraction and reverse transcription, section thickness, and fixation time. The pituitary glands were harvested using a pair of tweezers from the skulls and embedded in O. C. T. compound (Sakura Finetek, Tokyo, Japan), with immediate soaking in liquid nitrogen. The frozen tissues were cryosectioned at a thickness of 20 μm to be placed on PPS membrane slides and silane-coated glass slides (Muto Pure Chemicals, Tokyo, Japan).

### Ethanol fixation and NeuroTrace staining

The cryosectioned slices were fixed in 100% ethanol of molecular biology grade for 60 s and dried thoroughly using an electric dryer without heat for several minutes. The importance of tissue dehydration has previously been addressed [38]. We used an air dryer for this purpose. After immersion in the ethanol solution or after the NeuroTrace solution application, the color of tissue sections changes, and the original color were restored by airing with a dryer in about 5 min. The staining solution contained NeuroTrace 500/525 green fluorescent Nissl stain solution in DMSO (Invitrogen, CA, USA) diluted 1:30 in RNase-free water and RNasin Plus RNase Inhibitor (final concentration of 400 U/mL staining solution). Since it was suggested that inclusion of an RNase inhibitor in the staining solution was critical in protecting RNA from degradation [10], we added an appropriate concentration of RNase inhibitor in the staining solution, as reported in the literature. In the present study, RNasin Plus RNase Inhibitor (400 U/mL) was directly applied to the ROI of each section. The concentration of the RNase inhibitor can be adjusted to be 500–1,000 U/mL, depending on the sample size or tissue thickness. Approximately 200 μL of the solution was applied to each slide using a pipette or a syringe to cover all the slices and incubated for 60 s. Further, the staining solution was carefully removed using a pipette or a syringe, and the slices were dried thoroughly using an electric dryer, until the color of the tissue sections indicated complete dehydration. It took no more than 5 min for drying. NeuroTrace 500/525 green fluorescent Nissl stain was chosen as an optimal stain, because it was visualized clearly under the LMD7000 fluorescent light, i.e., LMD or LMG setting, whereas NeuroTrace 530/615 Red fluorescent was not suitable for this apparatus.

### Hematoxylin and eosin (H & E) staining for the pituitary gland

The sections on silane-coated glass slides were stained as follows: PBS for 10 s, Mayer’s hematoxylin (Muto Pure Chemicals) in aqueous solution for 30 s, DEPC-treated water for 5 s, eosin (Muto Pure Chemicals) in ethanolic solution for 30 s, followed by dehydration with 100% ethanol for 10 s, and Hemo-D (d-limonene; Falma, Tokyo, Japan) for 60 s.

### Storage

The sections on steel-framed PPS membrane slides for LMD were dried thoroughly and kept at room temperature, protected from light. After LMD, microdissected samples were stored in tightly sealed collection tubes and kept at room temperature. The present non-fixed and non-stained condition is the same condition as what has previously been proven to preserve RNA quality for several weeks [6]. No major RNA degradation was detected in the tissue sections on the PPS slides or the microdissected fragments during storage period, even at room temperature. The “1-day samples” denote that the microdissected samples were stored at room temperature overnight, followed by RNA extraction 20–24 h later. In the case of “8-day samples”, the microdissection was conducted within a week after fixation and staining, followed by RNA extraction on the 8th day. Prior to RNA extraction, collection tubes were centrifuged at 15,000×*g* for 1 min, to spin down the microdissected samples at the bottom of the collection tubes.

### RNA extraction and RT-qPCR

We examined RNA integrity by RT-qPCR based on a method described previously [6]. A solution containing RNA was obtained by dissolving LMD tissue samples in the CellAmp Direct RNA Prep Kit lysis buffer (Takara. Otsu, Japan) containing proteinase K (0.3 U, Takara), followed by incubation at 50°C for 30 min and then sonication for 1 min. Proteinase K was inactivated by incubation at 75°C for 5 min, followed by DNase treatment (0.05 U, Takara) at 37°C for 5 min. DNase was inactivated at 75°C for 5 min in a solution adjusted to an optimal concentration for the subsequent reverse transcription (PrimeScript, Takara) by adding 1.8-fold diluted EASY Dilution (Takara) which included approximately 10 ng of “carrier RNA”: 5′-GGACACAAGACAACAUAAAAAAAAAAAAAAAAAAAAAAAAA-3′. The “carrier RNA” design was based on chum-RNA described by Tougan et al. [42] and has no sequence homology to mouse cDNA. All aqueous solutions and reagents described above were adjusted as per the volume of the collected LMD samples. For all DG and pituitary samples having size below the 300,000 μm^2^ in area ×20 μm in thickness, a total of 21 μL of RNA-containing solution was prepared. The composition of each sample was: proteinase-K in lysis buffer in a 1:49 ratio (6 μL), DNase in lysis buffer in 1:9 ratio (1.5 μL), and 1.8-fold diluted EASY Dilution solution containing “carrier RNA” (13.5 μL). For pituitary samples exceeding the 300,000 μm^2^ area size, the amount of applied reagents were adjusted per 100,000 μm^2^ area size: proteinase-K in lysis buffer (2 μL), of DNase in lysis buffer (0.5 μL), and 1.8-fold diluted EASY Dilution solution (4.5 μL) per 100,000 μm^2^ area size.

Reverse transcription reactions were performed in a reaction mixture solution (30 μL), containing PrimerScript buffer (6 μL), oligo dT primer (1.5 μL), random N_6_ primers (1.5 μL), RT enzyme mix (1.5 μL), and of template from the RNA-containing solution (19.5 μL) described above. In addition, the reverse-transcription thermocycling parameters were as follows: 37°C for 30 min and 85°C for 5 s. The synthesized cDNA samples were transferred to less adsorbent tubes (e.g., Platinum super polypropylene BM4015, BMBio), and 2 μL was applied for each qPCR reaction.

Absolute transcript (rRNA and mRNA) levels were quantified by SYBR Green I-based qPCR using Thunderbird qPCR mix (Toyobo, Osaka, Japan) and a LightCycler instrument (Roche Molecular Biochemicals, Indianapolis, USA). Transcript levels are presented as copy number determined from a standard curve generated for each gene. For analyzing DG sections having size of 150,000 μm^2^ in area ×20–40 μm in thickness, the amount of cDNA template applied to qPCR reaction was calculated to commensurate with 9,286 μm^2^ × the specified cryosection thickness of microdissected tissue. On the other hand, for analyzing the pituitary gland samples, the transcript levels were normalized based on the GAPDH transcript levels due to the varying LMD sample sizes. The oligonucleotide primers used for the amplicons were as follows:

- 18S rRNA (*Fwd*): 5′-GGACCAGAGCGAAAGCATTTG-3′,
- 18S rRNA (*Rev*): 5′-TTGCCAGTCGGCATCGTTTAT-3′;
- GAPDH (*Fwd*): 5′-AACTTTGGCATTGTGGAAGG-3′,
- GAPDH (*Rev*): 5′-ACACATTGGGGGTAGGAACA-3′;
- β-actin (*Fwd*): 5′-AGCCATGTACGTAGCCATCC-3′,
- β-actin (*Rev*): 5′-CTCTCAGCTGTGGTGGTGAA-3′;
- Map2 (*Fwd*): 5′-CAGGATGACGGGCTGAAAT-3′,
- Map2 (*Rev*): 5′-GTGTGTGTGGAGAAGGGCAAC-3′;
- BDNF (*Fwd*): 5′-ATCGGCTTCACAGGAGACAT-3′,
- BDNF (*Rev*): 5′-TCAGGTCAACATAAACCACCA-3′;
- Arc (*Fwd*): 5′-CAGAGCCAGGAGAATGACAC-3′,
- Arc (*Rev*): 5′-GCAGCTTCAGGAGAAGAGAG-3′;
- cFos (*Fwd*): 5′-GAAGGGAACGGAATAAGATGG-3′,
- cFos (*Rev*): 5′-CTGTCTCCGCTTGGAGTGTA-3′.

### Assessment of the retained transcripts

To assess the degree of mRNA and rRNA yield due to degradation or washout during the fixation and staining procedures, an estimated amount of the retained transcripts was determined for each of the analyzed genes. It was calculated according to the following equation: ${\text{Retained transcripts}} (\% ) = 100 (t/\bar{T})$, where *t* is the transcript level (in copy number) of each treated sample and $\bar{T}$ is the mean transcript level (in copy number) of the untreated samples.

### Statistics and graphs

Values are expressed as mean ± standard error of the mean (SEM). Student’s *t* test, one-way ANOVA followed by Tukey post hoc test and two-way ANOVA followed by Bonferroni post hoc test were employed for statistical analysis using GraphPad Prism 5.0 (GraphPad Software, San Diego, USA) for Windows. The significance level between groups was defined as p < 0.05. Correlations between the transcript expression levels were determined by Pearson product-moment correlation coefficient, and graphs were generated using the GraphPad Prism 5.0 and Excel 2013.

## Additional files

Additional file: 1
**Figure S1.** Visualization of neurons of ethanol-fixed and NeuroTrace-stained third ventricle (D3V) specimen under (A_1_) a bright field and (A_2_) a fluorescence radiated field. The staining of the choroid plexus is thought to be a non-specific signal commonly observed in fresh frozen samples stained with regular Nissl stains, such as Cresyl violet, and is often considered negligible as it is irrelevant to the cerebral parenchyma. (A_3_) Ethanol-fixed hippocampal CA1 region under a fluorescent light, left side with the NeuroTrace stain and right side without it. (B_1_) Ethanol-fixed and NeuroTrace-stained neurons of the hippocampal DG region (B_2_) before and (B_3_) after microdissection, indicated by red arrows. Scales bars: (A) 200 μm, (B_1_) 100 μm, and (B_2_, B_3_) 25 μm. Additional file: 2
**Figure S2.** The hippocampal area in (A) unfixed and unstained, (B) ethanol-fixed, and (C) ethanol-fixed and NeuroTrace-stained tissues 12 days after the treatment. CA1 region in (D_1_) ethanol-fixed and NeuroTrace-stained tissue, and (D_2_) ethanol-unfixed and NeuroTrace-stained tissue. (E) Ethanol-fixed and NeuroTrace-stained tissue 30 days after the treatment. Scale bars, (A–C, E) 310 μm and (D) 100 μm. Additional file: 3
**Figure S3.** (A–C) Correlation of the transcript levels between the housekeeping genes in unfixed and unstained (Untreated; crosses) and ethanol and NeuroTrace-treated (EtOH/NT; circles) samples: (A) β-actin vs. 18S rRNA, (B) GAPDH vs. 18S rRNA, and (C) GAPDH vs. β-actin. (D–E) Correlation between the transcript levels of the housekeeping genes and Map2: (D) β-actin vs. Map2, (E) 18S rRNA vs. Map2, and (F) GAPDH vs. Map2. Values are expressed as copy number of transcripts per LMD tissue of 182 nm^2^ × cryosection thickness in volume. For untreated samples, *n* = 7 for 20 μm, *n* = 6 for 30 μm, and *n* = 8 for 40 μm; for fixed and stained samples, *n* = 7 for 20 μm, *n* = 7 for 30 μm, and *n* = 8 for 40 μm. Additional file: 4
**Figure S4.** Transcript levels of (A) 18S rRNA, (B) GAPDH, (C) β-actin, and (D) Map2 mRNAs in the hippocampal DG region from samples that were cryosectioned at a thickness of 20 μm, 30 μm, and 40 μm. Values are expressed as the copy number of genes per LMD tissue of 182 nm^2^ × cryosection thickness in volume. Bars indicate the mean ± SEM. Asterisks (**p* < 0.05, ***p* < 0.01, ****p* < 0.001) express statistically significant differences between fixed and stained specimens and the corresponding unfixed and unstained samples, as assessed by two-way ANOVA followed by Bonferroni post hoc test. For unfixed and unstained (Untreated) samples, *n* = 7 for 20 μm, *n* = 6 for 30 μm, and *n* = 8 for 40 μm; for fixed and stained samples, *n* = 7 for 20 μm, *n* = 7 for 30 μm, and *n* = 8 for 40 μm. Additional file: 5
**Figure S5.** Transcript levels of (A) 18S rRNA, (B) GAPDH, and (C) β-actin mRNAs in samples fixed with ethanol for 10 s, 30 s, and 60 s in comparison with the untreated samples. Values are expressed as copy number of transcripts per 182 nm^2^ × 20 μm cryosection thickness. Bars indicate mean ± SEM. One-way ANOVA followed by Tukey post hoc test, where ***p* < 0.01, ****p* < 0.001. *n* = 7 for the untreated samples, n = 6 for 10 s fixation, and n = 8 for 30 s and 60 s fixation. Additional file: 6
**Figure S6.** NeuroTrace-stained specimens (A) unfixed, under (A_1_) a bright field and under (A_2_) a fluorescence radiated field, (B) ethanol-fixed for 10 s, under (B_1_) a bright field and under (B_2_) a fluorescence radiated field, (C) ethanol-fixed for 30 s, under (C_1_) a bright field and under (C_2_) a fluorescence radiated field, and (D) ethanol-fixed for 60 s, under (D_1_) a bright field and under (D_2_) a fluorescence radiated field. Images were acquired on the day of fixation and staining. Scale bars, 200 μm.

## Abbreviations

LAM: laser-assisted microdissection
LMD: laser microdissection
LCM: laser capture microscopy
ROIs: regions of interest
DG: dentate gyrus
RT-qPCR: quantitative reverse transcription PCR
H & E: hematoxylin and eosin.
