# Supplementary figures and images for: Application of NeuroTrace staining in the fresh frozen brain samples to laser microdissection combined with quantitative RT-PCR analysis
Source: BMC Res Notes. 2015 Jun 20;8:252. doi: 10.1186/s13104-015-1222-9 (PMC4473849; doi:10.1186/s13104-015-1222-9)

Fig S1

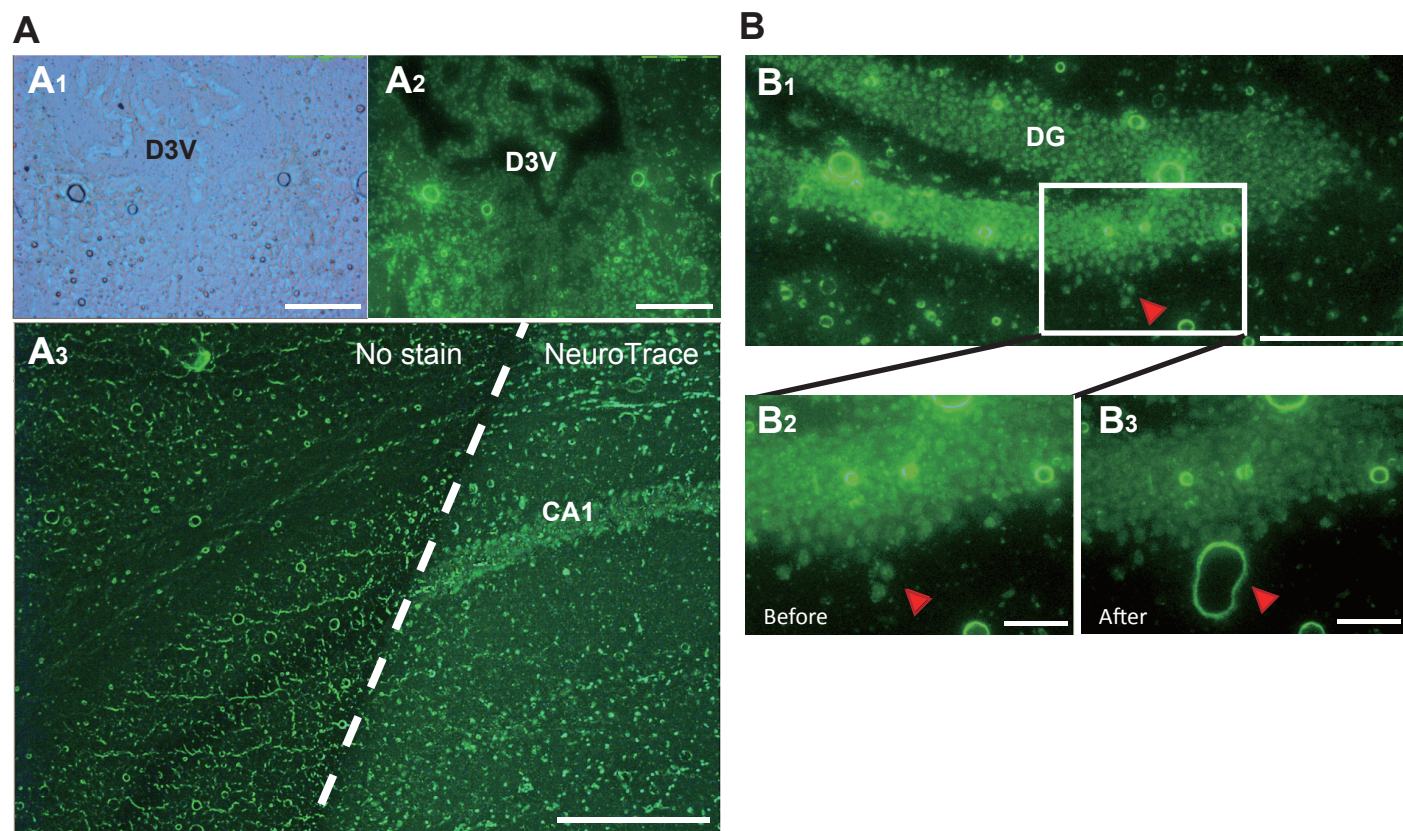

Supplement: Additional file: 1 — Figure S1. Visualization of neurons of ethanol-fixed and NeuroTrace-stained third ventricle (D3V) specimen under (A1) a bright field and (A2) a fluorescence radiated field. The staining of the choroid plexus is thought to be a non-specific signal commonly observed in fresh frozen samples stained with regular Nissl stains, such as Cresyl violet, and is often considered negligible as it is irrelevant to the cerebral parenchyma. (A3) Ethanol-fixed hippocampal CA1 region under a fluorescent light, left side with the NeuroTrace stain and right side without it. (B1) Ethanol-fixed and NeuroTrace-stained neurons of the hippocampal DG region (B2) before and (B3) after microdissection, indicated by red arrows. Scales bars: (A) 200 μm, (B1) 100 μm, and (B2, B3) 25 μm. [file 13104_2015_1222_MOESM1_ESM.pdf]

Fig. S2

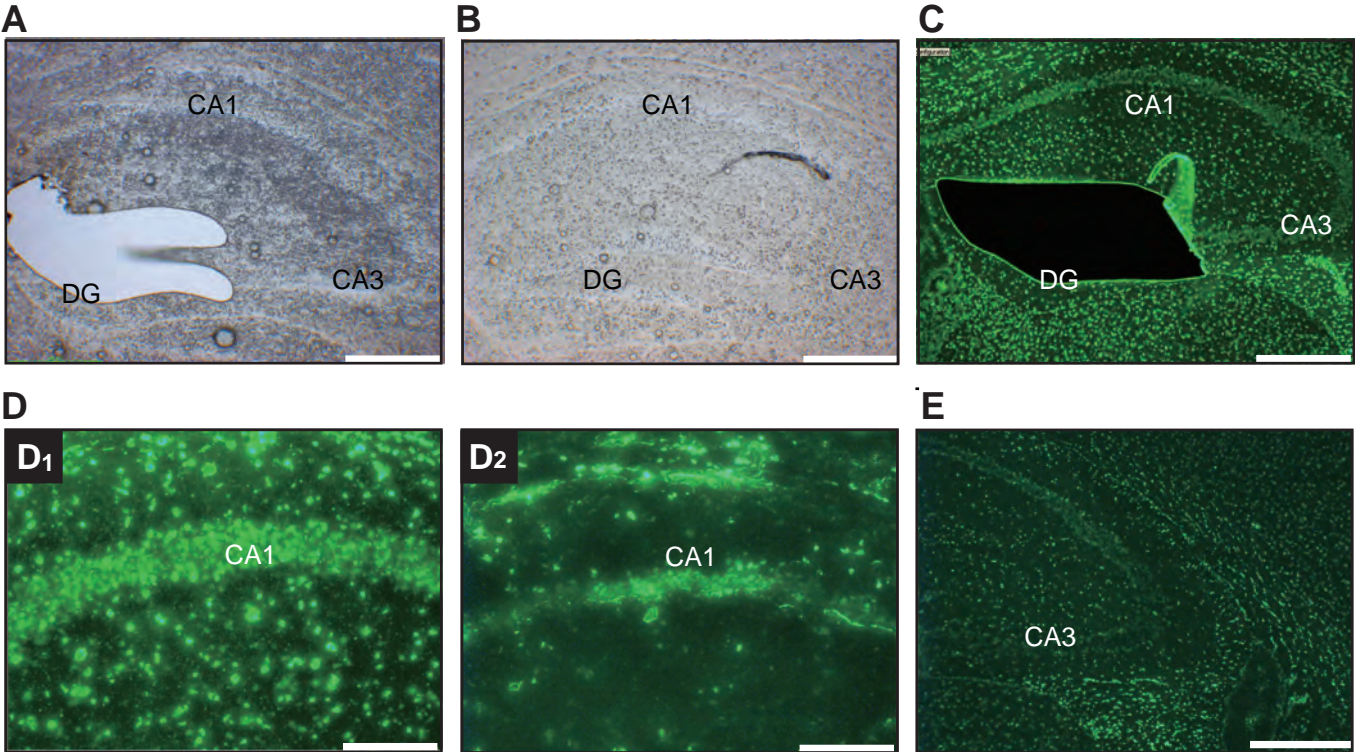

Supplement: Additional file: 2 — Figure S2. The hippocampal area in (A) unfixed and unstained, (B) ethanol-fixed, and (C) ethanol-fixed and NeuroTrace-stained tissues 12 days after the treatment. CA1 region in (D1) ethanol-fixed and NeuroTrace-stained tissue, and (D2) ethanol-unfixed and NeuroTrace-stained tissue. (E) Ethanol-fixed and NeuroTrace-stained tissue 30 days after the treatment. Scale bars, (A–C, E) 310 μm and (D) 100 μm. [file 13104_2015_1222_MOESM2_ESM.pdf]

Fig. S3

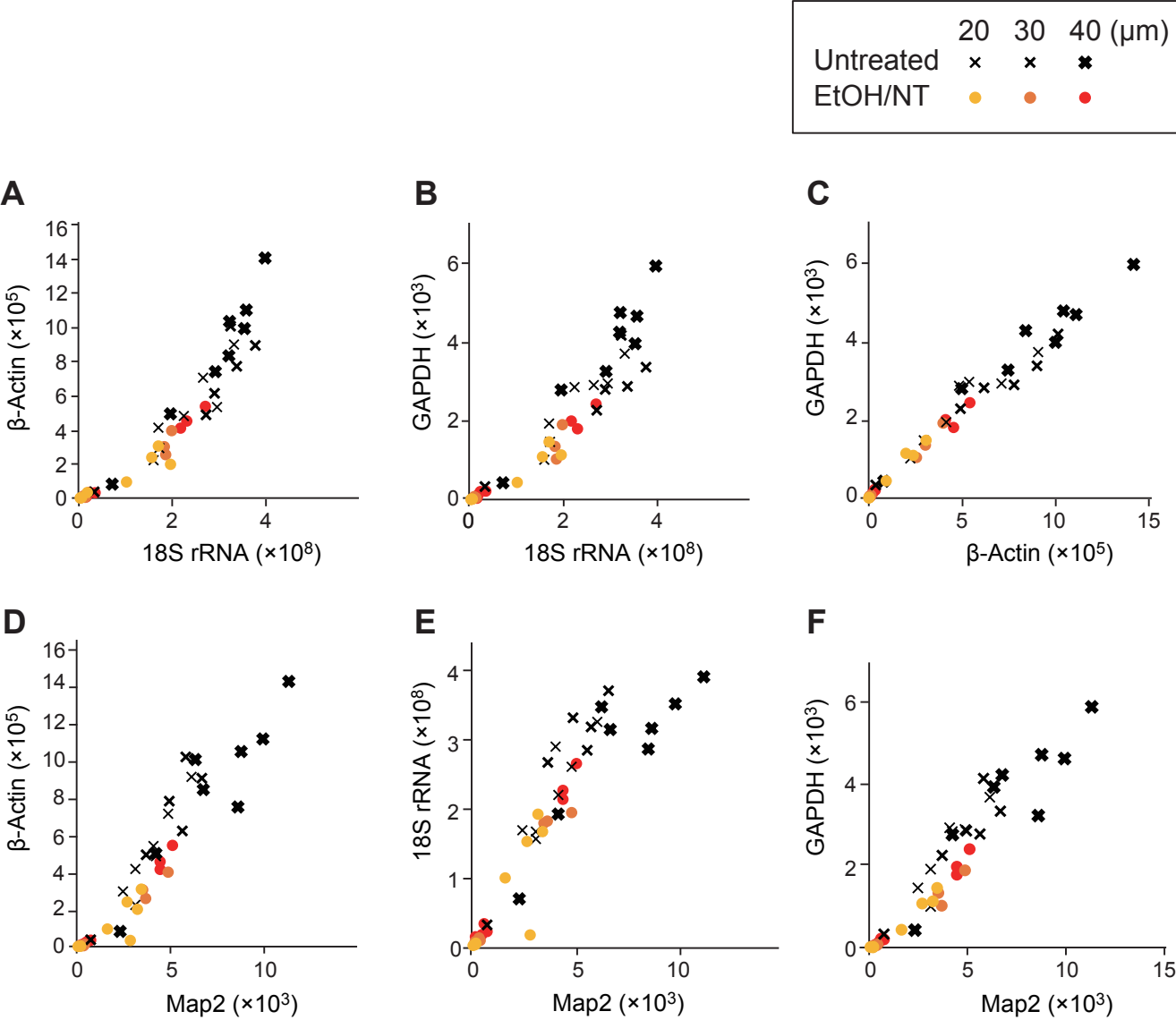

Supplement: Additional file: 3 — Figure S3. (A–C) Correlation of the transcript levels between the housekeeping genes in unfixed and unstained (Untreated; crosses) and ethanol and NeuroTrace-treated (EtOH/NT; circles) samples: (A) β-actin vs. 18S rRNA, (B) GAPDH vs. 18S rRNA, and (C) GAPDH vs. β-actin. (D–E) Correlation between the transcript levels of the housekeeping genes and Map2: (D) β-actin vs. Map2, (E) 18S rRNA vs. Map2, and (F) GAPDH vs. Map2. Values are expressed as copy number of transcripts per LMD tissue of 182 nm2 × cryosection thickness in volume. For untreated samples, n = 7 for 20 μm, n = 6 for 30 μm, and n = 8 for 40 μm; for fixed and stained samples, n = 7 for 20 μm, n = 7 for 30 μm, and n = 8 for 40 μm. [file 13104_2015_1222_MOESM3_ESM.pdf]

Fig. S4

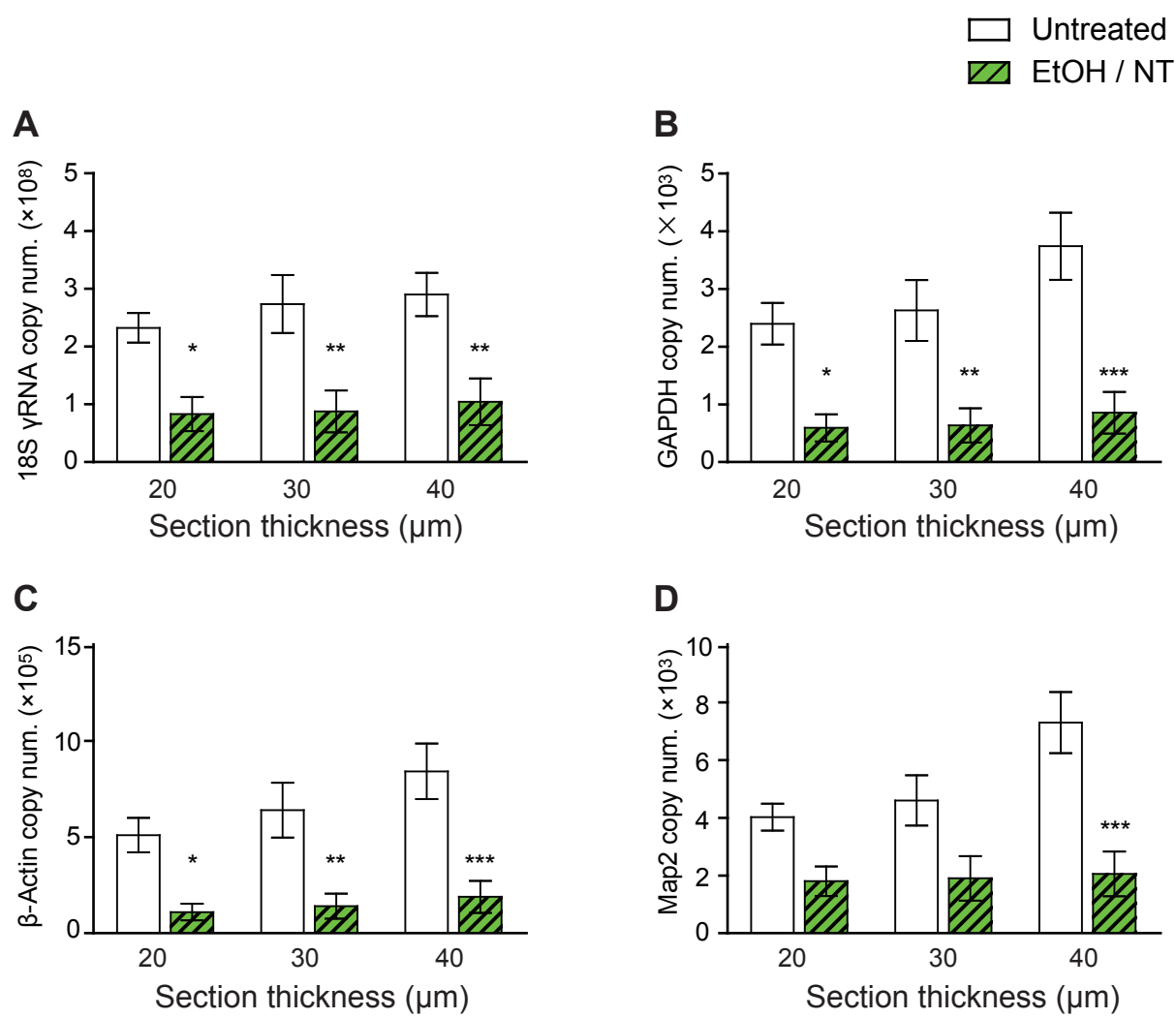

Supplement: Additional file: 4 — Figure S4. Transcript levels of (A) 18S rRNA, (B) GAPDH, (C) β-actin, and (D) Map2 mRNAs in the hippocampal DG region from samples that were cryosectioned at a thickness of 20 μm, 30 μm, and 40 μm. Values are expressed as the copy number of genes per LMD tissue of 182 nm2 × cryosection thickness in volume. Bars indicate the mean ± SEM. Asterisks (*p < 0.05, **p < 0.01, ***p < 0.001) express statistically significant differences between fixed and stained specimens and the corresponding unfixed and unstained samples, as assessed by two-way ANOVA followed by Bonferroni post hoc test. For unfixed and unstained (Untreated) samples, n = 7 for 20 μm, n = 6 for 30 μm, and n = 8 for 40 μm; for fixed and stained samples, n = 7 for 20 μm, n = 7 for 30 μm, and n = 8 for 40 μm. [file 13104_2015_1222_MOESM4_ESM.pdf]

Fig.S5

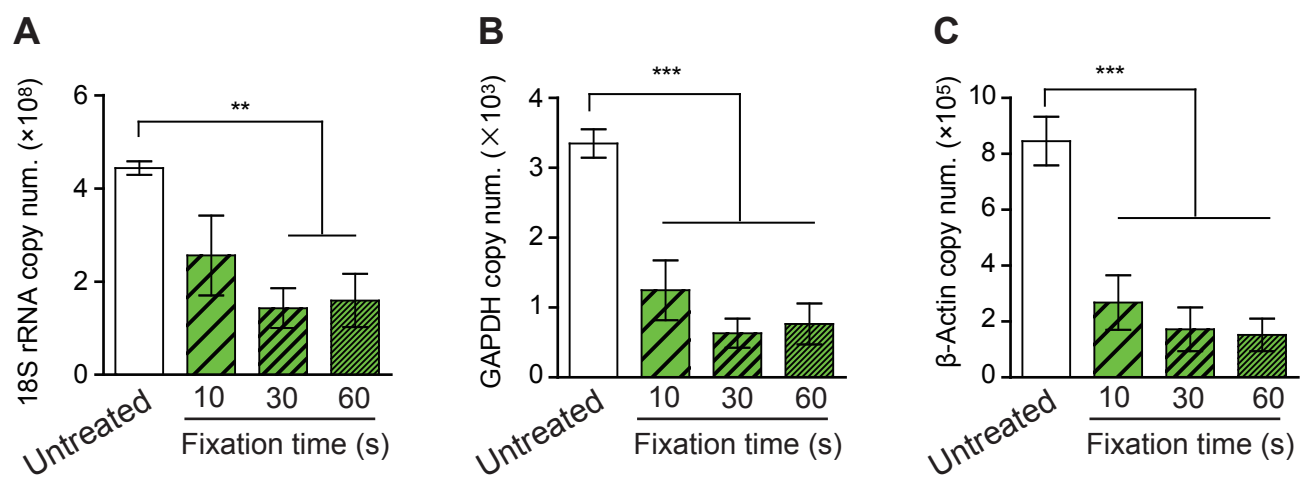

Supplement: Additional file: 5 — Figure S5. Transcript levels of (A) 18S rRNA, (B) GAPDH, and (C) β-actin mRNAs in samples fixed with ethanol for 10 s, 30 s, and 60 s in comparison with the untreated samples. Values are expressed as copy number of transcripts per 182 nm2 × 20 μm cryosection thickness. Bars indicate mean ± SEM. One-way ANOVA followed by Tukey post hoc test, where **p < 0.01, ***p < 0.001. n = 7 for the untreated samples, n = 6 for 10 s fixation, and n = 8 for 30 s and 60 s fixation. [file 13104_2015_1222_MOESM5_ESM.pdf]

Fig. S6

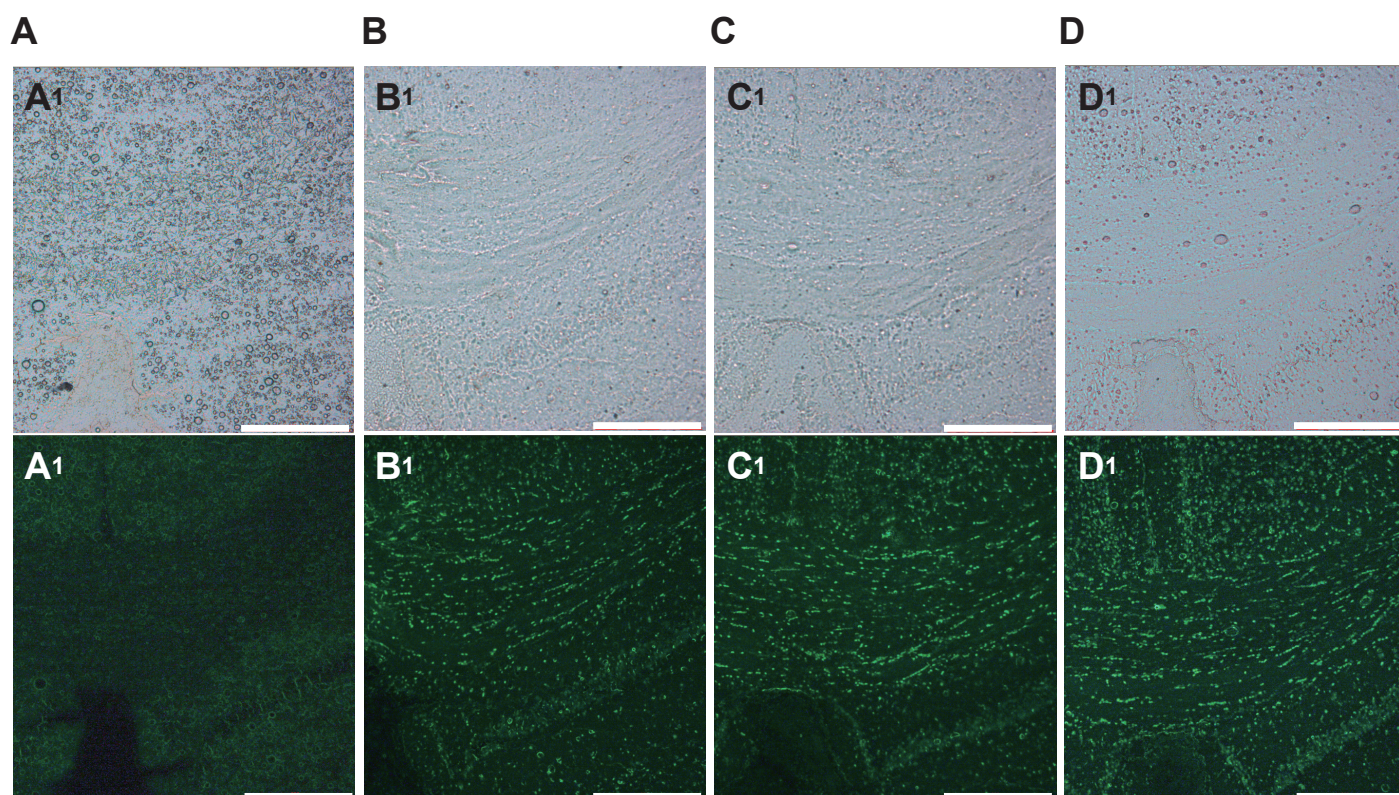

Supplement: Additional file: 6 — Figure S6. NeuroTrace-stained specimens (A) unfixed, under (A1) a bright field and under (A2) a fluorescence radiated field, (B) ethanol-fixed for 10 s, under (B1) a bright field and under (B2) a fluorescence radiated field, (C) ethanol-fixed for 30 s, under (C1) a bright field and under (C2) a fluorescence radiated field, and (D) ethanol-fixed for 60 s, under (D1) a bright field and under (D2) a fluorescence radiated field. Images were acquired on the day of fixation and staining. Scale bars, 200 μm. [file 13104_2015_1222_MOESM6_ESM.pdf]
